# In vitro evidence of bubble-induced acoustic softening and Sanal flow choking in cardiovascular decompression

**DOI:** 10.1038/s41526-025-00517-5

**Published:** 2025-08-11

**Authors:** V. R. Sanal Kumar, Pradeep Kumar Radhakrishnan, Dhruv Panchal, Dekkala Vinay, Yash Raj, Raunak Sharma, Yaman Vohra, Shivansh Rana, Sanjay Singh

**Affiliations:** 1 Amity Institute of Aerospace Engineering, Amity University Uttar Pradesh, Noida, India; 2 Biomexia India, Andhra University Innovation Hub, Visakhapatnam, Andhra Pradesh India; 3 Vyadh Aerospace, Ahmedabad, Gujarat India; 4 University of Queensland, Brisbane, QLD Australia; 5 University of Minnesota Twin Cities, Minneapolis, MN USA

**Keywords:** Biochemistry, Biophysics, Biotechnology, Neuroscience, Physiology, Biomarkers, Health care, Medical research, Neurology, Risk factors, Engineering, Biomedical engineering

## Abstract

When astronauts or divers experience a rapid drop in surrounding pressure, tiny gas bubbles can form in their blood—a condition that can threaten heart and vessel function. In this study, we simulated such decompression using fresh, warmed blood samples (37–40 °C) placed in a vacuum chamber. Bubbles consistently appeared near 600 mmHg. Their formation led to *acoustic softening*, a sharp drop in the speed of sound through blood. As flow velocity remained unchanged, the rising local Mach number brought the system closer to Sanal flow choking, triggered at a critical pressure ratio. Once choking occurs, it can lead to localized supersonic zones and abrupt pressure jumps. Additionally, bubbles may coalesce and block narrow vessels—a phenomenon akin to vapor lock—further impeding circulation. These findings reveal a novel mechanistic link between microbubble formation, acoustic softening, and flow choking, offering valuable insights for protecting cardiovascular health during spaceflight and rapid decompression events.

## Introduction

Decompression sickness (DCS)^1^ can occur when the surrounding pressure drops too quickly for dissolved gases in the body—mainly nitrogen—to safely remain in solution, leading to bubble formation in blood and tissues. While it is often explained by gas buildup alone, clinical observations such as sudden heart problems and uneven neurological symptoms suggest that other physical mechanisms may also be involved. A long-standing simplification in cardiovascular modeling is to treat blood as incompressible, a convenient assumption that permits straightforward pressure–flow calculations^2–5^. Recent thermodynamic studies show that no flowing fluid can ever be truly incompressible^6–8^. For a fluid to behave that way, two key heat-related properties—called specific heats at constant pressure and constant volume—would have to be equal, which is physically impossible because it would violate the basic laws of energy conservation^8^. Under rapid decompression, high acceleration, or microgravity, blood’s finite compressibility can no longer be ignored. Although various in vitro, in silico, and in vivo studies have explored Sanal flow choking under compressible multiphase (gas–plasma–suspension) flow conditions^6–20^, the specific role of *acoustic softening* caused by microbubble nucleation in triggering Sanal flow choking has not been previously reported.

When ambient pressure falls, tiny gas nuclei can grow into microbubbles in the bloodstream. Even a void fraction of only 0.5–1% lowers the speed at which sound travels through blood—a phenomenon called *acoustic softening* first described by Wood’s mixture equation^21^. Because the bulk flow velocity stays the same while acoustic velocity falls, the local Mach number (flow speed divided by sound speed) rises sharply. As it approaches one, conditions favor Sanal Flow Choking (SFC)—a high-speed-flow effect in which the mass passing a narrowing becomes fixed and shock-like pressure jumps appear just downstream. In a narrowed (stenosed) artery—hydrodynamically akin to a converging–diverging (CD) nozzle—these jumps can damage the vessel lining (endothelial integrity) and disturb the heart’s own blood supply (myocardial perfusion) at a critical pressure ratio^22,23^.

Microbubbles add a second hazard by coalescing into a “vapor lock,” physically blocking or slowing blood in tight vessels and magnifying pressure surges on already-stressed arterial walls. A third, subtler risk emerges when a bubble ruptures. Once the pressure drop across the breach reaches the critical total-to-static pressure ratio defined by SFC theory^6^, the escaping gas initially chokes at the opening, reaching sonic speed. The surrounding streamlines then flare outward, acting like the divergent section of a tiny CD nozzle. This geometry allows the jet to accelerate into a pocket of supersonic flow, which inevitably terminates in a microscopic shock wave where it re-enters the subsonic bloodstream. These miniature shocks impose additional mechanical stress on the endothelium and can trigger cascading injury. Together—acoustic softening, vapor-lock obstruction, and supersonic-jet shocks from bubble rupture—these interconnected phenomena reveal a compounded cardiovascular threat, especially during rapid decompression, high-altitude flight, or prolonged exposure to microgravity.

When dissolved gases nucleate into microbubbles during decompression, they do more than just form visible bubbles—they fundamentally alter the acoustic properties of blood. According to Wood’s equation^21^, even a small amount of gas (a void fraction of just 0.5–1%) can reduce the speed of sound in blood from ~1500 m/s to below 100 m/s. This dramatic drop occurs within milliseconds of bubble onset and increases the local Mach number (flow velocity relative to sound speed), pushing blood flow closer to critical choking conditions. This phenomenon, known as *acoustic softening*, is not only a key early marker of decompression stress but also a precursor to more severe flow disturbances in the cardiovascular system^22–32^.

Surface properties play a crucial role in where and when bubbles form. Heterogeneous nucleation theory states that rough or creviced surfaces—like those found on stents or aged vascular walls—require lower pressure drops to initiate bubble growth^33,34^. Notably, progressive vascular aging and the cumulative effects of minor flow choking and shock-like events may gradually roughen the arterial lumen^22–27^, further increasing the risk of microbubble nucleation due to enhanced surface friction. Our in-house in vitro experiments^35–37^ confirm that threaded stent analogs trigger bubble formation significantly earlier than smooth ones, consistent with the classic crevice nucleation model proposed by Atchley and Prosperetti^33,34^. These findings highlight the importance of surface engineering in medical devices intended for high-risk environments such as spaceflight or deep-sea missions, where decompression events are more likely.

SFC is a localized phenomenon in fluid dynamics wherein, upon reaching a critical ratio of total to static pressure, the flow attains sonic conditions^6–8^. This condition can arise even at moderate flow velocities, leading to a constant mass flow rate and sudden pressure drops downstream. These shock-like pressure fronts, though microscopic, exert intense mechanical stress on already compromised vascular regions, such as stenosed arteries. To experimentally validate these conditions, we exposed freshly drawn venous blood to decompression profiles simulating both spacecraft cabin pressure loss and rapid repressurization during extravehicular activity (EVA) suit operations. By integrating compressible-flow theory with cardiovascular biomechanics, this study introduces a new paradigm—bio-shock-wave mechanics—linking aerospace propulsion principles to human physiology. Understanding and predicting SFC under these conditions is vital for improving risk models, refining protective strategies, and ensuring cardiovascular resilience in extreme operational settings.

## Results and discussion

**Fig. 1:**
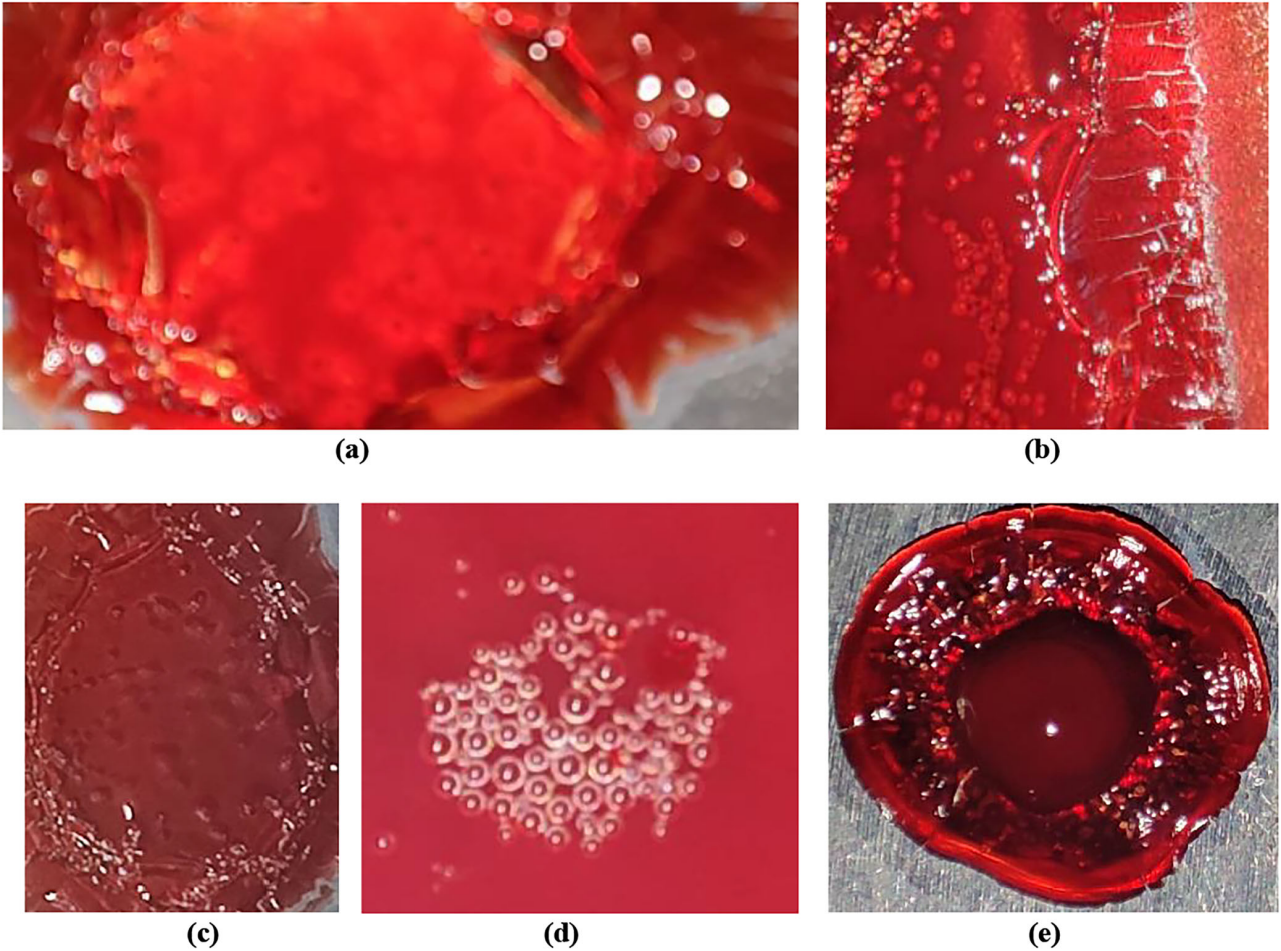
Microbubbles in venous blood of healthy humans under reduced pressure. **a**–**e** Magnified views of microbubble nucleation and associated textural changes in venous blood samples collected from different healthy adult individuals, exposed to vacuum-induced decompression (350–650 mmHg at 40 °C). All images depict visually detectable microbubble formation, with notable inter-subject variation in bubble distribution, density, and structural transitions. These variations reflect individual-specific thermophysical responses of blood under decompressive stress, offering insight into the morphological pathways leading to potential cardiovascular risks in extreme environments such as spaceflight.

**Fig. 2:**
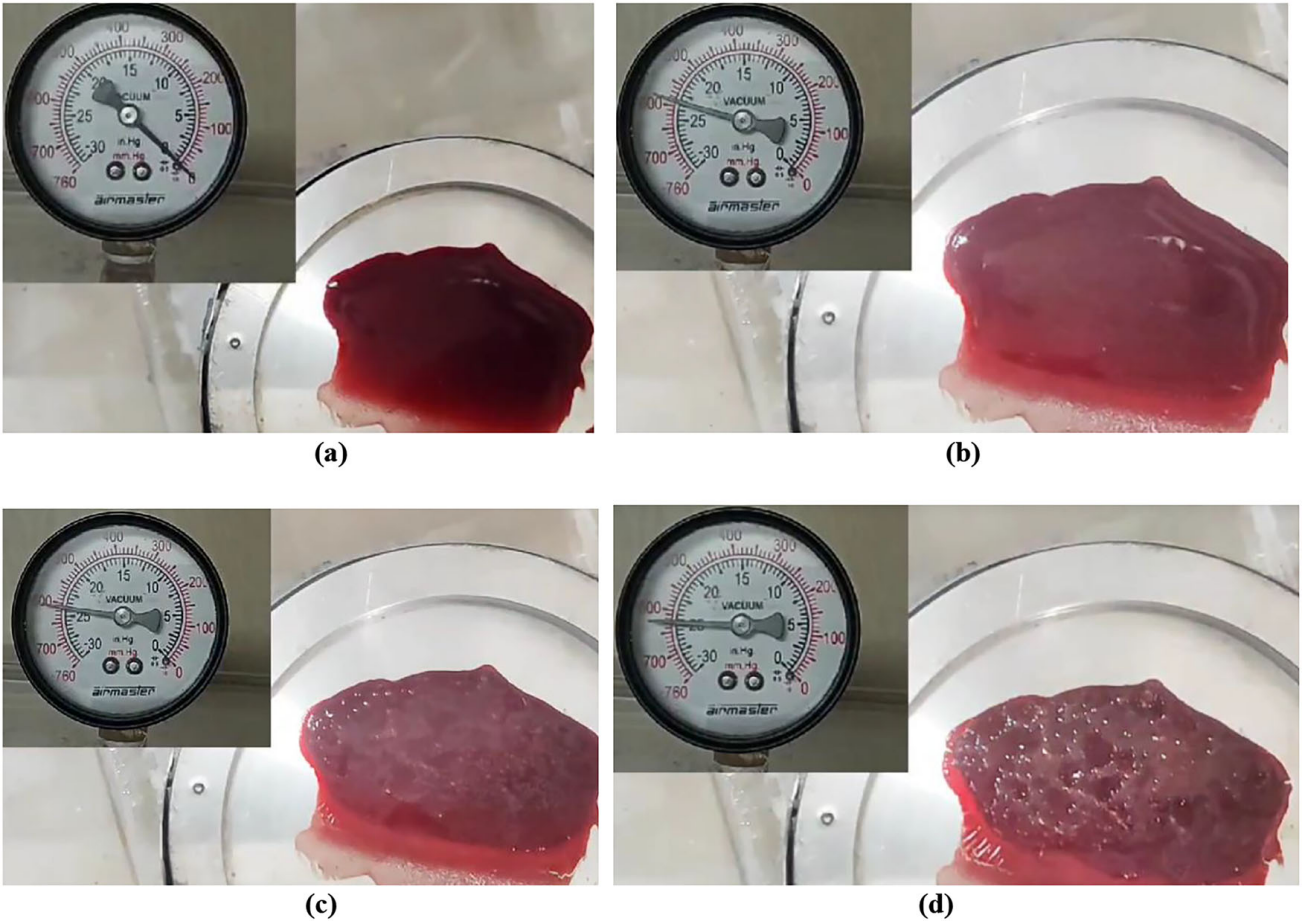
Subject-specific differences in microbubble formation under vacuum-induced decompression. **a**–**d** Progressive stages of visually detectable bubble nucleation and phase transitions in venous blood during vacuum-induced decompression at 40 °C, showing: **a** fresh blood sample before devacuumization, **b** initial observable bubbling at 590 mmHg, **c** increased bubble intensity at 610 mmHg, and **d** advanced aggregation and structural transitions at 625 mmHg.

This pilot in vitro study explored how decompression-induced microbubble nucleation and acoustic softening contribute to flow instability and potential SFC in human blood. The experimental setup simulated pressure conditions relevant to DCS, especially during high-altitude exposure and spaceflight, offering new insights into the compressible behavior of blood under stress. A visual sequence captured during the experiment demonstrated progressive microbubble nucleation and distinct phase transitions in venous blood as it was subjected to vacuum-induced decompression ranging from 350 to 650 mmHg at 40 °C (see Figs. 1, 2). Prior to the vapor pressure test, the blood sample appeared stable, while post-test observations revealed significant gas evolution and structural changes. A video of the full vapor pressure test is available at: https://www.youtube.com/watch?v=pHStIayAYas.

### Bubble nucleation and acoustic softening–induced Sanal flow choking

Gradual decompression of fresh venous blood samples at physiological temperatures (37–40 °C) led to reproducible microbubble formation, which first became visible between 590 and 625 mmHg [Fig. 2a–d]. As pressure continued to decrease, nucleation intensified, suggesting the presence of a vapor pressure threshold that initiates bubble formation. Repeated trials confirmed consistent nucleation behavior at pressures below ~170 mmHg. Surface texture was found to play a key role in bubble formation: rough-threaded surfaces promoted earlier and denser bubble nucleation compared to smooth ones, supporting classical crevice-nucleation theory^26^. Preliminary observations also suggested subtle gender-based differences in nucleation dynamics, though further investigation is required to validate this finding^38^.

**Fig. 3:**
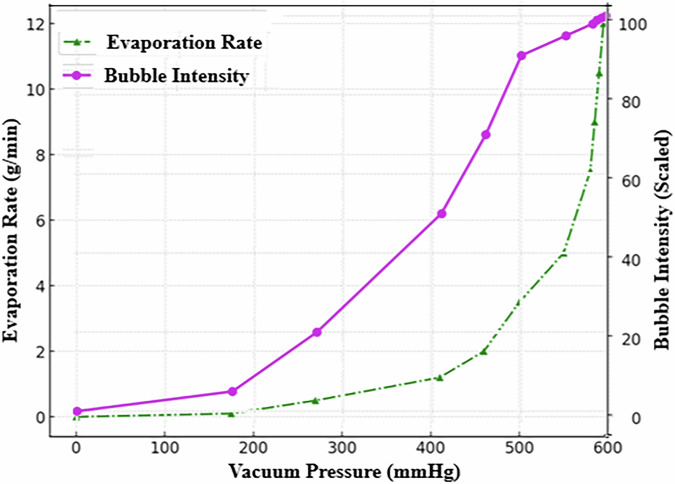
Blood evaporation rate and bubble formation intensity in a 26-year-old healthy male (O+ blood type) during vacuum-induced decompression at 40 °C. The dual-axis plot shows the relationship between vacuum pressure (mmHg) and two key responses: evaporation rate (g/min, green dashed line with triangles) and scaled bubble formation intensity (magenta solid line with circles). Both parameters exhibit nonlinear increases as ambient pressure decreases, with a pronounced rise in activity between 500 and 600 mmHg. This critical pressure range marks the onset of phase transitions and microbubble nucleation, offering insights into decompression-induced cardiovascular risks.

High-speed imaging revealed a series of progressive thermal and phase changes in blood during decompression. Initial plasma outgassing was followed by increasingly vigorous bubbling, indicating evaporation. At more extreme pressure drops, visual signs of protein denaturation and polymerization emerged, including crust formation at the sample edges. Ultimately, near-complete moisture loss led to a brittle, coagulated state, mimicking tissue behavior under severe decompression conditions. These results (Figs. 1–3) illustrate a clear sequence of decompression-induced phase transitions, with potential relevance to the structural and functional integrity of blood under extreme environmental stress.

To validate chamber performance and benchmark fluid behavior, parallel decompression experiments were conducted using water and hydrocarbon fuels. Water drawn from the Narmada River exhibited expected boiling behavior between 545 and 560 mmHg, confirming the reliability of the vacuum decompression system. Narmada water was selected due to its moderate mineral content and regional availability. While this freshwater sample served to validate the experimental setup, future comparisons using seawater—given its higher salinity and dissolved gas content—may offer additional insights into pressure-dependent nucleation behavior.

Petrol, diesel, and kerosene displayed nucleation patterns consistent with their thermophysical properties^35–37,39^. Petrol, with the highest vapor pressure, nucleated earliest, while kerosene exhibited the greatest resistance to bubble formation. These control tests affirmed that fluids with lower heat capacity and higher vapor pressure are more susceptible to early phase transitions under decompression. These findings supported the experimental framework used to investigate blood behavior and provided a reference for interpreting nucleation onset and thermal responses.

Microgravity and space radiation are among the most critical environmental stressors encountered during space missions, with well-documented effects on cellular function, fluid dynamics, and vascular physiology. However, opportunities to study these effects directly in space remain limited due to high mission costs and constraints on experimental repeatability. To address this, ground-based microgravity simulators have been widely used to replicate altered gravity conditions and examine biological responses at the cellular and systemic levels^40^. Complementing these approaches, the present study introduces a vacuum-based test protocol to simulate another key aspect of spaceflight: pressure variation and decompression stress, which can significantly influence blood behavior. By demonstrating how microbubble nucleation, acoustic softening, and SFC^6^ occur under controlled vacuum conditions, our findings offer a novel and physiologically relevant method for assessing cardiovascular risks associated with spaceflight-induced decompression events—providing an important bridge between microgravity simulation and in situ physiological modeling.

The study confirmed that the phenomenon of SFC^6,7^ in cardiovascular system is closely associated with a reduction in sound velocity in bubbly blood flows. As gas bubbles form and disperse, they decrease the speed of sound, leading to compressibility effects and potential shock wave generation. Oscillating bubbles further dissipate energy, reducing sound speed, particularly at resonance frequencies. When gas concentration increases (see Fig. 3), the sound velocity in blood may approach the velocity of blood flow itself, leading to shock wave formation and cardiovascular disruptions such as embolism and hemorrhage, especially in extreme environments like space travel or rapid decompression scenarios.

The observations from this study have significant implications for understanding the risks associated with DCS and cardiovascular health in extreme environments. The formation and behavior of microbubbles under reduced pressure conditions can lead to flow choking, shock wave generation and potential cardiovascular disruptions. Understanding these mechanisms is crucial for developing effective decompression protocols and protective measures for individuals exposed to rapid pressure changes, such as divers and astronauts.

The study corroborates SFC as a novel concept in cardiovascular physiology^6^, at different physical situations, demonstrating that when the internal-to-external pressure ratio of tiny bubbles exceeds a critical threshold, their rupture can trigger streamtube expansion, leading to significant shock wave formation. This suggests that blood’s compressibility under extreme conditions plays a pivotal role in the development of DCS and other cardiovascular disorders. Furthermore, the study confirms that fluids with higher vapor pressures and lower heat capacities are more susceptible to early nucleation under decompression. This finding underscores the importance of understanding the thermophysical properties of biofluids in assessing the risks associated with rapid decompression scenarios.

This pilot investigation confirms, on first-law grounds, that every moving fluid—including blood—is inherently compressible, because its enthalpy necessarily exceeds its internal energy; therefore, haemodynamic studies that aim to capture SFC must incorporate compressible-flow effects, especially under decompression. Here we demonstrate that a rapid pressure drop initiates a coupled cascade of microbubble nucleation, acoustic softening, and SFC in whole blood. Reproducible vacuum-gauge thresholds marked a sequence from plasma outgassing through evaporation and protein polymerization to final coagulation, and high-speed imaging revealed transient shock-like fronts whenever local Mach numbers approached unity. Rough-textured coupons triggered earlier, denser bubbling than polished controls, underscoring surface micro-geometry as a key design variable for future cardiovascular devices.

Although absolute sound speed was inferred rather than measured, the void-fraction–dependent softening predicted by Wood’s equation aligns with the observed flow perturbations and offers a mechanistic bridge between nitrogen supersaturation and the abrupt vascular events reported in DCS. The parallel tests in water, petrol, diesel, and kerosene validate our pressure-ramp protocol and situate blood within a wider thermophysical landscape. These findings establish a tractable bench model—*bio-shock-wave mechanics*—for exploring how pressure transients in spaceflight, high-altitude aviation, or deep-sea ascent can compromise endothelial integrity, myocardial perfusion, and overall haemodynamic stability. They also point to practical countermeasures: smoother implant surfaces, revised prebreathe schedules, and real-time monitoring of acoustic properties during EVA.

Future work should (i) directly measure sound speed and pressure fields in pulsatile, endothelialised flow systems, (ii) map blood heat capacity and vapor pressure across diverse clinical cohorts to refine flow choking thresholds, and (iii) integrate high-fidelity bubble metrics into AI-driven wearables for early risk prediction. Addressing these gaps will convert the present proof-of-concept into an operational toolset for safeguarding cardiovascular health in extreme environments.

This pilot investigation has several limitations that future work should address. First, acoustic softening was inferred rather than measured; direct, high-fidelity quantification of sound speed, void fraction, and bubble kinematics is needed. Techniques such as digital inline holography and three-dimensional particle-tracking velocimetry could capture bubble size, shape, density, and trajectories, while high-speed confocal or phase-contrast microscopy would reveal membrane thickness, rupture modes, and wall adhesion. Complementary laser-induced fluorescence and Raman spectroscopy could identify the evolving gas composition inside and around bubbles.

Real-time mapping of void fraction and microbubble intensity is another critical gap. X-ray phase-contrast imaging, optical coherence tomography, and ultrasound backscatter analysis can supply high-resolution distributions of microbubbles in flowing blood analogs or vascular constructs, generating richer datasets for AI-driven decompression-risk models.

Quantifying the exact pressure ratio that triggers SFC in anatomical geometries will require pressure-sensitive microchannels fitted with micro-PIV and embedded MEMS pressure sensors. Such instrumentation can pinpoint the threshold at which choking and shock-like jumps begin.

Bubble nucleation is also highly sensitive to surface microtexture and radiation-induced changes in material chemistry. Atomic-force microscopy and three-dimensional profilometry should be used to characterize roughness, whereas controlled irradiation studies can reveal how space-relevant radiation alters surfaces, blood chemistry, membrane stability, and thermophysical properties that influence nucleation and choking.

An integrated, AI-ready data pipeline is essential. Future experiments must systematically capture bubble morphology, growth kinetics, spatial void-fraction fields, gas composition, surface correlations, critical pressure ratios, Mach-number evolution, and the roles of temperature, viscosity, heat capacity, and geometry under variable pressure, radiation, and microgravity conditions.

Ultimately, real-time, in-vivo monitoring will depend on miniaturized optical, acoustic, or magneto-elastic sensors capable of detecting microbubble onset, acoustic-property shifts, local pressure gradients, and velocity changes indicative of choking. Coupling these data streams to adaptive AI models that reference individual physiological baselines could give astronauts early warning of decompression stress, enabling preventive action before symptomatic DCS or cardiovascular compromise develops. Addressing these limitations with advanced diagnostics will advance a real-time cardiovascular-risk surveillance framework for deep-space missions, uniting experimental fluid physics, bioengineering, and artificial intelligence to safeguard crew health.

## Methods

This in vitro pilot study investigated bubble nucleation and SFC behavior in blood under decompression using a custom-built vacuum chamber system. Ethics approval is not required for this research involving non-invasive blood sample testing from healthy adult volunteers, as no patient identifiers or clinical interventions were involved. All participants provided informed consent in accordance with institutional and Government of India guidelines.

### Blood sample testing

Venous blood from healthy adult volunteers was placed in a temperature-controlled decompression chamber and subjected to controlled vacuum (760 to 100 mmHg) while maintaining physiological temperatures (37–40 °C). Microbubble nucleation events were documented using time-resolved imaging with a high-frame-rate mobile camera, and bubble intensity was quantified. The chamber pressure was gradually reduced from atmospheric pressure (~760 mmHg) to ~100 mmHg under monitored conditions. The experimental setup included multiple pressure gauges, gas/smoke detectors, a thermostatic temperature controller (maintaining 37–40 °C), and a video imaging system to monitor bubble nucleation, phase transition, and thermal effects during decompression.

The onset of bubble nucleation, vapor pressure thresholds, and related morphological changes in blood samples were systematically documented. High-resolution imaging allowed detailed visualization of microbubble behavior and progressive protein denaturation during decompression. A distinct sequence of phase transitions was observed across the vacuum gauge range: initial plasma outgassing with minor bubbling occurred between 0 and 270 mmHg; more pronounced bubbling between 410 and 500 mmHg indicated plasma evaporation and early protein denaturation; crust formation associated with protein polymerization was noted between 550 and 580 mmHg; and complete coagulation and solidification followed at 585–595 mmHg. These transitions closely resemble physiological conditions associated with DCS. The findings support that gas supersaturation in blood precedes visible bubble formation, with nucleation initiated as ambient pressure drops below critical thresholds.

The experiments were carried out at Vyadh Aerospace, Ahmedabad, and Amity University, India, in accordance with the ethical standards set by the Government of India for research involving healthy adult volunteers. As part of the sanctioned DST-Amity-TEC project, all procedures for this in vitro study adhered to applicable national and institutional regulations. The experimental protocols were reviewed and approved by both the Head of Research and Innovation and the Director of the Amity Institute of Aerospace Engineering, Amity University Uttar Pradesh. Blood samples were obtained from healthy adult volunteers at the Amity Clinic, following a formal request by the Principal Investigator of the DST-Amity-TEC project and after obtaining informed consent from all participants. All necessary approvals for conducting blood-based experiments on healthy individuals were secured in compliance with institutional and governmental guidelines.

## Data Availability

The data that support the findings of this study are available within the article.

## Code availability

This study did not involve any software code or computational tools. All data were generated through in vitro method.
